# Nervous and Mental Diseases

**Published:** 1900-04

**Authors:** 


NERVOUS AND MENTAL DISEASES. A Manual for Students and Practitioners. By CHARLES S. POTTS, M. D., Instructor in Nervous Diseases, University of Pennsylvania. LEA'S SERIES OF POCKET TEXT-BOOKS. Edited by Bern B. Gallaudet. M. D., Illustrated with 88 engravings. Philadelphia and New York: Lea Brothers & Co.

The author does not profess to place before the profession a work that will supersede the more complete text-books on neurology—but simply one that will contain all that is necessary for a working knowledge of nervous and mental diseases. It is a concise exposition of the most modern knowledge of the two closely allied subjects and will find favor among those who have not the time to delve into the larger and more complete works. The author does not take up the anatomy and histology of the nervous system to any extent—just simply enough to freshen one's memory upon points that are essential to the proper understanding of many of the pathological conditions and the symptoms arising therefrom.

The first few chapters are devoted to the symptomatic disorders, the diseases of the peripheral nerves, of the cranial nerves, spinal nerves and meninges. The system diseases have been classified according to the neuron or neurons affected. Thus, locomotor ataxia is a disease confined usually to the sensory tract, in some cases, however, motor neurons may also suffer. Progressive muscular atrophy is a disease of the motor neurons, acute anterior paleomylitis is described under the peripheral motor neurons, and Frederick's ataxia under the combined motor and sensory neurons. The author states that it may not be strictly accurate owing to the complicated nature of the subject, yet it is believed to be as nearly so as the present state of knowledge permits. These diseases have always been considered as being motor or sensory in character, long before the neuron theory was projected on the neurological curtain and will no doubt always remain where they were originally placed, neuron theory or no neuron theory.

A few chapters are devoted to mental diseases, not enough to do much good, but just enough to make the enquiring student ask for more.

Full attention is given to matters of general symptomatology and methods of examination, and therapeutic measures both medicinal and non-medicinal, such as electricity, massage, hydrotherapeutics, and rest cure, are thoroughly discussed in the light of the most recent discoveries and achievements.

Although especially useful for students this work will prove of great value to practitioners, posting them thoroughly in the most recent points regarding a class of diseases which are exceedingly common and important, and often puzzling.

The illustrations are well chosen and numerous.

This work is similar in appearance to Lea's Pocket Text-book series and makes a beautiful addition to the library shelf.

